# A unique array of neuroprotective effects of pyruvate in neuropathology

**DOI:** 10.3389/fnins.2015.00017

**Published:** 2015-02-17

**Authors:** Yuri Zilberter, Olena Gubkina, Anton I. Ivanov

**Affiliations:** Institut de Neurosciences des Systèmes, Aix Marseille Université, Inserm UMR_S 1106Marseille, France

**Keywords:** energy metabolism, pyruvate, neurodegeneration, oxidative stress, PARP-1, NAD, neuroinflammation

The three common signature characteristics of many neurological diseases are brain hypometabolism, oxidative stress, and neuroinflammation (Melo et al., 2011; Cai et al., 2012; Heneka et al., 2014). In order to be efficient, successful treatment should target all three pathologies simultaneously. Pyruvate seems to be an ideal candidate for such a treatment because of its unique combination of neuroprotective effects (Figure 1). In this opinion paper, we attempt to review and summarize recent information concerning these effects and their significance for neuroprotection.

**Figure 1:**
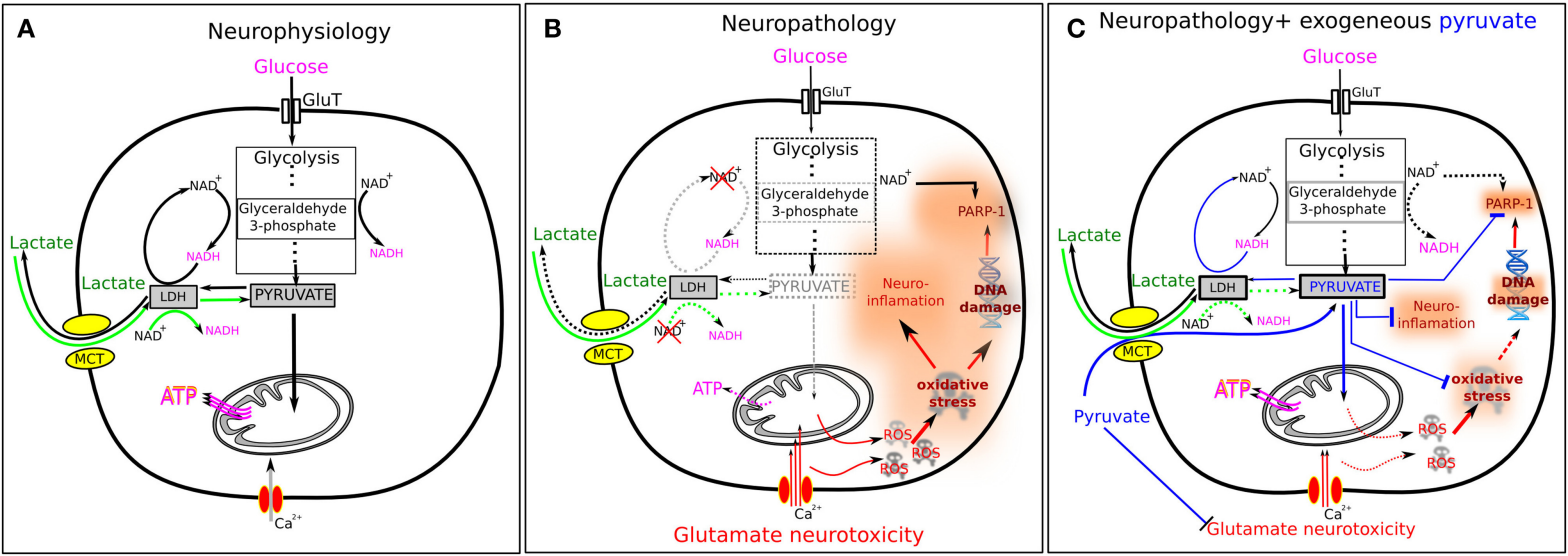
**Pyruvate and lactate in normal and pathological conditions**. **(A)** In normal conditions, glucose enters the cell via glucose transporters (GluT) and is metabolized in a 10-step glycolysis. Endogenous pyruvate is a final product of glycolysis and the main energy substrate for ATP generation in mitochondria. Excessive for mitochondria pyruvate can be transformed to lactate by lactate dehydrogenase (LDH) in the presence of NADH. Extracellular lactate can enter the cell via monocarboxylate transporters (MCT). Lactate then is converted to pyruvate by LDH in the presence of NAD^+^ (green arrows). This explains why extracellular lactate may serve efficiently as the energy fuel for brain cells. **(B)** The situation is changed radically under pathological conditions. A number of neurological disorders are characterized by the oxidative stress and increased level of interstitial glutamate—both factors inducing strong excitotoxicity. Oxidative stress results from excessive presence of ROS, while glutamate induces cellular overload with Ca^2+^ ions. ROS induce DNA damage leading to the overactivation of poly-ADP ribose polymerase-1 (PARP-1) that results in depletion of cytosolic NAD^+^. ROS also activate the pro-inflammatory transcription factor NF-kB inducing neuroinflammation. Depletion of NAD^+^ induces inhibition of glycolysis since the glycolysis step 6, conversion of glyceraldehyde 3-phosphate to 3-phosphoglycerate, requires two molecules of NAD^+^. This results in the insufficient outcome of pyruvate and decline in the mitochondrial ATP production. Moreover, NAD^+^ depletion makes ineffectual the conversion of lactate to pyruvate (dashed green arrows) and lactate cannot serve as the energy substrate anymore. **(C)** Exogenous pyruvate is able to ameliorate many impaired cellular functions described in **(B)**. In blood, pyruvate activates a blood resident enzyme glutamate–pyruvate transaminase which transforms glutamate into 2-ketoglutarate and thus lowers the blood glutamate level. This results in an enhanced efflux of glutamate from brain parenchyma that reduces neuronal overload with Ca^2+^ ions. Pyruvate reacts directly with H_2_O_2_ producing acetate, H_2_O and CO_2_ and thus reducing oxidative stress. Pyruvate inhibits PARP-1 overactivation that prevents depletion of NAD^+^ and thus promotes glycolysis (glyceraldehyde-3-phosphate depending step). It also inhibits expression of several pro-inflammatory proteins, such as tumor necrosis factor (TNF), interleukin 6 (IL-6) and others. All these effects explain the neuroprotective properties of pyruvate.

## Pyruvate enhances the brain-to-blood glutamate efflux

Perisynaptic astrocytes normally provide fast take-up of glutamate released during synaptic activity. In pathological conditions however, extracellular glutamate levels can be abnormally high and neurotoxic (Wang and Qin, 2010). Part of this glutamate can be cleared via glutamate transporters located in the capillary endothelial cells that form the blood-brain-barrier. The efficacy of such efflux depends on the glutamate concentration gradient between blood and interstitial fluid (Teichberg et al., 2009). Meanwhile, blood glutamate content can be lowered by activation of a blood-resident enzyme glutamate–pyruvate transaminase that in the presence of pyruvate transforms glutamate into 2-ketoglutarate (Gottlieb et al., 2003), thus reducing the glutamate blood concentration. This should favor the glutamate flux from the interstitial fluid to the blood. Therefore, extracellular glutamate levels can be controlled in part by blood pyruvate, which can enhance the brain-to-blood glutamate efflux.

Indeed, Zlotnik and co-authors demonstrated (Zlotnik et al., 2008, 2012) that intravenous injection of pyruvate after traumatic brain injury in rats led to a transient decrease in blood glutamate levels and significantly improved neurological outcome during the first days following injury as well as hippocampal neuron survival at 30 days after injury.

One of the most severe acute neurological conditions, associated with excessive glutamate release, is the status epilepticus (SE). Glutamate-induced excitotoxicity is largely based on massive influx of Ca^2+^ via glutamate receptors, which seems to be a necessary step in the overall process of neuronal degeneration and the acute neuronal cell death that occurs after SE. Morphological analysis of the rat brain after pilocarpine-induced SE demonstrates that the hippocampal subfield CA1 and the hilus of dentate gyrus are particularly susceptible to neuronal cell loss. SE-induced neuronal loss in CA1 was largely prevented in rats treated with pyruvate plus oxaloacetate (i.p. injection 30 min after development of SE) (Carvalho et al., 2011). Moreover, neuronal damage in the dentate gyrus was prevented in rats that received pyruvate alone while oxaloacetate alone did not reveal any neuroprotective effects. The authors related the observed beneficial effects to the blood glutamate scavenging, although other capabilities of pyruvate could also influence the positive outcome.

## Pyruvate non-enzymatically scavenges H_2_O_2_

Pyruvate in relatively small concentrations (<1 mM) protects neurons against H_2_O_2_–induced toxicity (Desagher et al., 1997). This effect is not related to the pyruvate's function as an energy substrate but rather to its ability to non-enzymatically interact with H_2_O_2_ producing acetate, water, and carbon dioxide (Holleman, 1904). The antioxidant effects of pyruvate and other alpha-ketoacids has been confirmed both *in vitro* in several cell types including neurons and *in vivo* in whole organs such as heart or kidney (Desagher et al., 1997; Das, 2006). Accumulation of reactive oxygen species (ROS) is a prominent feature of oxidative stress and by scavenging ROS pyruvate may substantially reduce the toxic consequences of this pathological event.

## Anti-inflammatory action of pyruvate

Many studies on different organs provided evidence that pyruvate (ethyl pyruvate) is an effective anti-inflammatory agent (reviewed in Kao and Fink, 2010). They demonstrated that pyruvate treatment down-regulates activation of the pro-inflammatory transcription factor, NF-kB, as well as the expression of several pro-inflammatory proteins, such as tumor necrosis factor (TNF), interleukin 6 (IL-6) and others (Das, 2006; Kao and Fink, 2010). The mechanism of this pyruvate effect is yet unclear although it may be explained, at least partly, by the pyruvate antioxidant properties as well as by the pyruvate-induced inhibition of poly-ADP ribose polymerase-1 (PARP-1) overactivation (see below and Figure 1C).

## Pyruvate enhances glycogen content in astrocytes

Pyruvate supplementation prior to glucose deprivation significantly protected synaptic function against the deleterious effects of hypoglycemia in brain slices (Shetty et al., 2012). The authors associated beneficial effect of pyruvate with both increased glycogen content during pyruvate pretreatment and subsequent glycogen utilization during glucose deprivation leading to the increased ATP levels. Interestingly, both extra glucose and lactate pretreatment also increased the glycogen content, although significantly less efficiently than pyruvate. However, neither lactate nor extra glucose pretreatment was sufficient to provide the protective effect on synaptic transmission during glucose deprivation.

Pyruvate chronic supplementation also strongly increased the glycogen content of cortical tissue *in vivo* in the Alzheimer's disease mouse model (APPswe/PS1dE9) (Zilberter et al., 2013).

## Pyruvate provides neuroprotection against damage induced by Poly-ADP ribose polymerase-1 overactivation

Poly-ADP ribose polymerase 1 (PARP-1) synthesizes polymers of ADP-ribose that are implicated in regulation of a number of cellular processes including modulation of transcription, DNA repair, neuronal survival and death (Smith et al., 2013). Importantly, to generate polymers of ADP-ribose PARP-1 consumes cytoplasmic NAD^+^. In various neurological disorders, excessive activation of PARP-1 by oxidative stress has been documented (Ma et al., 2012). This process compromised cell survival via activation of pro-death pathways by ADP-ribose polymers and by creating energy deficit via depletion of cytoplasmic NAD^+^ that was followed by inhibition of glycolysis and ATP production (see Figure 1B).

It has been also reported recently that PARP-1 directly inhibits hexokinase (Andrabi et al., 2014), increasing its potential for blocking glycolysis. Importantly, Ying and colleagues reported (Ying et al., 2002) that exogenous TCA cycle substrates (including pyruvate) administration following PARP-1 activation reduced cell death in the astrocyte–neuron cultures from approximately 70% to 30%.

Similar neuroprotective effects of pyruvate was reported *in vivo* in transient cerebral ischemia and severe hypoglycemia models, in which PARP-1 had been shown to be a key mediator of neurotoxicity (Suh et al., 2003; Moroni and Chiarugi, 2009). In these models, pyruvate treatment either completely prevented the neuronal loss or reduced it by 70–90% (Lee et al., 2001; Suh et al., 2005). Brain damage reduction due to pyruvate treatment was also reported in the rodent model of traumatic brain injury with documented prominent oxidative stress, PARP-1 overactivation and loss of NAD^+^ (Satchell et al., 2003; Clark et al., 2007; Fukushima et al., 2009; Sharma et al., 2009). Venous infusion of pyruvate after controlled arterial hemorrhage in swine reduced oxidative stress and PARP fragmentation in the brain (Mongan et al., 2003). Although elucidating the exact mechanisms of pyruvate neuroprotection was beyond the scope of these studies, the authors suggested that the pyruvate action includes the ROS scavenging, NAD^+^ replenishment, recovering the pyruvate-dehydrogenase activity and direct mitochondrial fueling.

Interestingly, PARP-1 overactivation was also demonstrated in the brain of transgenic Alzheimer's disease mouse model (Abeti et al., 2011). In mixed cultures of neurons and glial cells, β-amyloid peptide, the major neurotoxic agent in the pathophysiology of Alzheimer's disease, evokes oxidative stress followed by hyperactivation of PARP-1, depolarization of mitochondrial membrane and finally cell death. (Abeti and Duchen, 2012). Addition of pyruvate to culture medium of β-amyloid treated cells prevented the mitochondrial membrane potential loss (Abramov and Duchen, 2005) and improved cell survival (Alvarez et al., 2003).

One reasonable explanation for the efficient pyruvate action may be in its antioxidant properties. Since PARP-1 is activated in response to oxidative damage to DNA, reducing oxidative stress would decrease PARP-1 activity resulting in NAD^+^ depletion. In addition, exogenous pyruvate can provide energy in conditions when glycolysis intensity is reduced due to a low cytoplasmic NAD^+^. Indeed, pyruvate is a “direct” energy substrate for mitochondria, while lactate needs to be converted first to pyruvate in the reaction dependent on the availability of cytoplasmic NAD^+^. Importantly, mitochondrial pool of NAD^+^, indispensible for pyruvate metabolism in mitochondria, is maintained for at least 24 h when cytoplasmic NAD^+^ is depleted (Stein and Imai, 2012), thus ensuring energy production.

## Antiepileptic effects of pyruvate

Recently, a robust antiepileptic effect of pyruvate (combined with antioxidants ascorbic acid and alpha-tocopherol) treatment has been revealed in the genetic model of temporal lobe epilepsy (Simeone et al., 2014). In addition, the authors showed that a single pretreatment of wild-type mice with these drugs reduced the severity of kainate-induced events resulting in 100% protection from severe tonic–clonic seizures. Unfortunately, the authors did not determine the contribution of each applied drug to the antiepileptic effect. To the best of our knowledge neither ascorbic acid nor alpha-tocopherol expresses significant antiepileptic action (Waldbaum and Patel, 2010). Therefore, we believe that pyruvate is the major player in the Simeone's work and the pyruvate antiepeileptic effect is presumably reinforced by complementary antioxidants.

Neuronal hyperactivity leading to abnormal oscillations and epilepsy, characteristic for Alzheimer's disease (Amatniek et al., 2006; Noebels, 2011), has been observed as well in different mouse models of the disease (Palop and Mucke, 2009). We found (Minkeviciene et al., 2009; Zilberter et al., 2013) that the general reason for hyperactivity may be the Aβ-induced modification of basic neuronal properties, such as the resting membrane potential and reversal potential of GABA-induced currents, presumably evoked by energy metabolism imbalance. Critically, in the presence of pyruvate, Aβ failed to induce its deleterious effects on the cellular parameters. Moreover, pyruvate chronic dietary supplementation considerably reduced epileptic phenotype in APP/PS1 mice (Zilberter et al., 2013). In another Alzheimer's disease model (3xTg-AD mice), chronic pyruvate treatment reduced both oxidative stress and hyperexcitability, and inhibited short and long-term memory deficits (Isopi et al., 2014).

## Conclusions

Oxidative stress and metabolic dysfunction are significant pathogenic factors contributing to neurological disorders. Pyruvate may be a unique therapeutic tool for correcting neuronal network abnormalities developing due to these factors. Combination of the following properties validates this conclusion: (i) Oxidative stress is the general feature of neurological disorders and is associated with accumulation of ROS. Pyruvate is a potent scavenger of ROS and its contribution to the antioxidant defense system becomes significant during neuropathologies; (ii) The oxidative stress-induced overactivation of PARP-1 results in the depletion of cytosolic NAD^+^ and inhibition of glycolysis that evokes energy deficiency and frequently results in a cell death. Pyruvate significantly abates overactivation of PARP-1. In addition, as pyruvate is a direct substrate for mitochondrial metabolism and its oxidation does not depend on the cytoplasmic redox state, pyruvate bypasses restrictions imposed by PARP-1 and can restore energy deficiency in such conditions; (iii) Pyruvate reduces the blood glutamate level, facilitating the glutamate efflux from brain tissue through the blood-brain barrier thus reducing the glutamate-induced neurotoxicity; (iv) Pyruvate augements glycogen stores, thus increasing neuronal tolerance to ischemia and hypoglycemia; (v) Neuroinflammation is a common attribute of a number of neuropathologies. Pyruvate reveals a potent anti-inflammatory action; (vi) Pyruvate prevents neural network hyperexcitability.

We conclude that pyruvate, in addition to its well-recognized function in energy metabolism, is a powerful neuroprotector, the potential therapeutic significance of which is yet widely underrated.

### Conflict of interest statement

The authors declare that the research was conducted in the absence of any commercial or financial relationships that could be construed as a potential conflict of interest.
